# Integration of single-cell RNA-seq and bulk RNA-seq data to construct and validate a cancer-associated fibroblast-related prognostic signature for patients with ovarian cancer

**DOI:** 10.1186/s13048-024-01399-z

**Published:** 2024-04-16

**Authors:** Liang Shen, Aihua Li, Jing Cui, Haixia Liu, Shiqian Zhang

**Affiliations:** 1 Department of Obstetrics and Gynecology, Liaocheng People’s Hospital, 67 Dongchang West Road, Liaocheng, Shandong 252000 P.R. China; 2 Shandong University, Jinan, P.R. China; 3 Department of Gynecology, Shandong Provincial Hospital Affiliated to Shandong First Medical University, 324 Jingwuweiqi Road, Jinan, Shandong 250021 P.R. China; 4 Department of Oral and Maxillofacial Surgery, Jinan Stomatology Hospital, 101 Jingliu Road, Jinan, Shandong 250001 P.R. China; 5 Central Laboratory of Jinan Stamotological Hospital, Jinan Key Laboratory of Oral Tissue Regeneration, 101 Jingliu Road, Jinan, Shandong 250001 P.R. China; 6 Department of Obstetrics, Shandong Provincial Hospital Affiliated to Shandong First Medical University, 324 Jingwuweiqi Road, Jinan, Shandong 250021 P.R. China; 7 Department of Obstetrics and Gynecology, Qilu Hospital of Shandong University, Jinan, Shandong 250012 P.R. China

**Keywords:** Cancer-associated fibroblasts, Ovarian cancer, Tumor microenvironment, Immune therapy

## Abstract

**Background:**

To establish a prognostic risk profile for ovarian cancer (OC) patients based on cancer-associated fibroblasts (CAFs) and gain a comprehensive understanding of their role in OC progression, prognosis, and therapeutic efficacy.

**Methods:**

Data on OC single-cell RNA sequencing (scRNA-seq) and total RNA-seq were collected from the GEO and TCGA databases. Seurat R program was used to analyze scRNA-seq data and identify CAFs clusters corresponding to CAFs markers. Differential expression analysis was performed on the TCGA dataset to identify prognostic genes. A CAF-associated risk signature was designed using Lasso regression and combined with clinicopathological variables to develop a nomogram. Functional enrichment and the immune landscape were also analyzed.

**Results:**

Five CAFs clusters were identified in OC using scRNA-seq data, and 2 were significantly associated with OC prognosis. Seven genes were selected to develop a CAF-based risk signature, primarily associated with 28 pathways. The signature was a key independent predictor of OC prognosis and relevant in predicting the results of immunotherapy interventions. A novel nomogram combining CAF-based risk and disease stage was developed to predict OC prognosis.

**Conclusion:**

The study highlights the importance of CAFs in OC progression and suggests potential for innovative treatment strategies. A CAF-based risk signature provides a highly accurate prediction of the prognosis of OC patients, and the developed nomogram shows promising results in predicting the OC prognosis.

**Supplementary Information:**

The online version contains supplementary material available at 10.1186/s13048-024-01399-z.

## Introduction

Ovarian cancer (OC) is one of the most prominent and potentially lethal gynecological malignancies, with a reported 313,959 newly diagnosed cases and 207,252 fatalities globally in 2020 [1]. Due to the lack of a mechanism for early detection and specific early-warning symptoms, OC patients are often diagnosed at an advanced stage, resulting in a 5-year survival rate of only 47% [2]. Although conventional platinum-based chemotherapeutic agents and cytoreductive resection can achieve complete remission, the majority of patients will eventually develop treatment resistance [3]. Immunotherapy has made significant advances in the last two decades and has ushered in a new era in the treatment of various cancers [4]. Although the success rate of OC immunotherapy remains unsatisfactory, the use of immune-checkpoint inhibitors (ICIs), chimeric antigen receptor (CAR), and T cell receptor-engineered T cells is advancing rapidly [5].

The tumor microenvironment (TME) has recently been found to have an instrumental function in the carcinogenesis of OC [6]. A specialized subset of fibroblasts called cancer-associated fibroblasts (CAFs) performs a critical function in the microenvironment of solid tumors, where they can modulate cancer progression and metastasis [7]. It has been demonstrated that CAFs can promote cancer progression by secreting growth factors, cytokines, and chemokines, and by degrading the extracellular matrix (ECM) [8–10]. CAFs have also been shown to generate prometastatic cytokines in a paracrine manner, thereby facilitating the metastasis of OC cells [11]. Moreover, CAFs contribute to immune evasion by upregulating of immune checkpoint ligands and immunosuppressive cytokines, hindering the infiltration of anti-tumor CD8 + T lymphocytes and provoking an anti-tumor response through interaction with other immune cells [12]. Increasing evidence suggests that CAFs mediate chemoresistance in OC [13]. Therefore, CAFs represent a promising therapeutic target for the treatment of OC [14].

The study aimed to fill a gap in our understanding of the role of CAFs in OC and to investigate their potential as a prognostic biomarker and therapeutic target. By analyzing scRNA-seq and transcriptomic data, the researchers could identify CAFs subclusters and develop a risk signature that was predictive of prognosis in OC patients. They also explored the relationship between the risk signature and the immune landscape of the tumor microenvironment and found that it was predictive of response to immunotherapy. Finally, the researchers developed a nomogram that integrated the CAF-based signature with other variables to aid in predicting the prognosis of OC patients in clinical settings.

## Materials and methods

### Data assembly and processing

Gene Expression Omnibus (GEO) data set GSE184880 was obtained, which contained scRNA-seq information from seven OC samples and five ovarian tissues. Initially, single cells were assessed to ensure that each gene was expressed in at least three cells and that each cell expressed a minimum of 250 genes to generate scRNA-seq data. The percentages of mitochondria and rRNA were subsequently calculated utilizing the PercentageFeatureSet tool in the Seurat R package. Additional screening was performed on the single cells by setting them to express a minimum of 6,000 genes with UMI > 100. Finally, 40,810 cells were retained. From The Cancer Genome Atlas (TCGA), we retrieved the transcriptomic data, copy number variations (CNV) data of the Masked Copy Number Segment, single-nucleotide variant (SNV) data, and relevant OC clinical data. We accessed the OV project of the TCGA database (http://cancergenome.nih.gov/) to extract the transcriptomes and clinical data of 379 OC patients, whereas the information on 88 normal tissues was gathered from the GTEx database. Patients without adequate clinical data were excluded from the analyses. After excluding samples of normal tissue and tumors derived from the GEO database lacking data on follow-up and outcomes, the GSE140082 cohort containing 380 OC samples was retrieved for use as a validation dataset. The literature was searched for ten cancer-associated pathways, including HIPPO, TP53, NOTCH, PI3K, TGF-Beta, RAS, NRF1, WNT, MYC, and Cell Cycle [15].

### Definition of CAFs

We performed a re-analysis of the OC scRNA-seq data with the aid of the Seurat program [16] to characterize the CAFs’ signature fully. The first step was to exclude the cells that had either > 5000 or < 250 expressed genes, which was accompanied by log normalization of these genes. Next, the uniform various approximation and projection approach was used for the non-linear dimensionality reduction, with 15 principal components and a resolution of 0.1. With the use of the FindNeighbors and FindClusters functions, single cells were organized into a variety of distinct subgroups (dim = 30 and resolution = 0.1). Afterward, the RunTSNE function was adopted to execute t-distributed stochastic neighbor embedding (TSNE) dimensionality reduction. The four marker genes, namely ACTA2, FAP, PDGFRB, and NOTCH3 were identified as being specifically expressed in fibroblasts and were annotated accordingly. The FindClusters and FindNeighbors tools from the original method were employed to re-cluster the fibroblasts. Clusters of fibroblasts were then subjected to dimensionality reduction using TSNE. With the FindAllMarkers tool, marker genes for each CAFs cluster were determined by performing pairwise comparisons across clusters based on an adjusted p-value < 0.05, minpct = 0.35, and logFC = 0.5. Employing the clusterProfiler program, an enrichment analysis was completed with the Kyoto Encyclopedia of Genes and Genomes (KEGG) on the CAF clusters’ marker genes [17]. Subsequently, the CopyKAT in R program was utilized to discriminate between tumors, and normal cells present per sample by analyzing the CNV features across the CAFs clusters [18].

### Discovery of CAFs-related hub genes

The limma program was employed to search for differentially expressed genes (DEGs) between tumor tissues and normal tissues with a |log2(Fold change) |>1 and false discovery rate (FDR) < 0.05 [19]. We next used Pearson’s correlation to determine which DEGs were most strongly associated with each CAF cluster. We used this information to determine the most important genes involved in CAF with *p* < 0.05 and cor > 0.4. In addition, genes associated with prognosis were discovered via univariate Cox regression analysis in the survival package at a p-value < 0.05. We conducted a least absolute shrinkage and selection operator (Lasso) cox regression analysis to minimize the number of genes, after which a multivariate Cox regression analysis was conducted using a stepwise regression technique. The equation below describes the risk signature generated from the output values of the multivariate Cox model: risk score=$\sum _{i=1}^{n}(\text{b}\text{i} \times \text{E}\text{x}\text{p}\text{i})$, whereby the risk signature gene is denoted by i, the expression profile of gene i is denoted by Expi, and bi denotes the gene i coefficients in the multivariate model. Zero-mean normalization was then performed to classify the patients into low- and high-risk categories. The timeROC program was utilized to conduct a receiver operating characteristic (ROC) analysis of the risk signature’s prediction accuracy. The validation cohort was also subjected to similar analyses.

### Development of a risk signature and nomogram

To develop a nomogram model suitable for clinical application, we initially conducted univariate and multivariate analyses on clinicopathological and risk signature parameters. With the rms program, the factors in the multivariate model with a p-value of < 0.05 were utilized to construct a nomogram for estimating the prognosis of OC individuals [20]. The generation of the calibration curve aided in evaluating of the model’s ability to make accurate predictions. Decision curve analysis (DCA) was conducted to assess the model’s reliability.

### Immune landscape analysis

The CIBERSORT algorithm [21], a method for evaluating immune cell infiltration, was employed to probe the distributions of 22 subtypes of immunocytes in the TCGA cohort. To additionally evaluate the TME, we used the ESTIMATE method to calculate stromal and immune scores [22].

### Analysis of genetic mutations associated with CAFs

The “maftools” R software was utilized for the purpose of constructing the genomic landscape of CAF-associated genes with SNV and CNV from the TCGA datasets.

### Prediction of immunotherapy sensitivity

To investigate the direct predictive value of the risk score on PD-1 therapy response, we utilized the IMvigor210 cohort, which comprised 298 patients with urothelial carcinoma and included transcriptomic data along with treatment response to immunotherapy [23].

### Validation using data in the cancer cell line encyclopedia (CCLE)

To validate the markers at the cellular level, we retrieved the mRNA expression patterns of those markers in 41 fibroblasts and 69 OC cell lines from the CCLE platform (https://portals.broadinstitute.org/ccle) [24]. We used a heat map to analyze the differences in the expression of these markers between fibroblasts and OC cell lines.

### Statistical analysis

The R software (v4.2.2) was applied to conduct all statistical data analyses. Pearson or Spearman correlations were utilized to create the matrices of correlation. All pairwise comparisons between the two groups were made via the Wilcoxon test. K-M curves and the Log-rank test were conducted to evaluate the significance of survival differences. The significance criterion was established at *P* < 0.05.

## Result

### Evaluation of CAFs in scRNA-seq samples

**Fig. 1:**
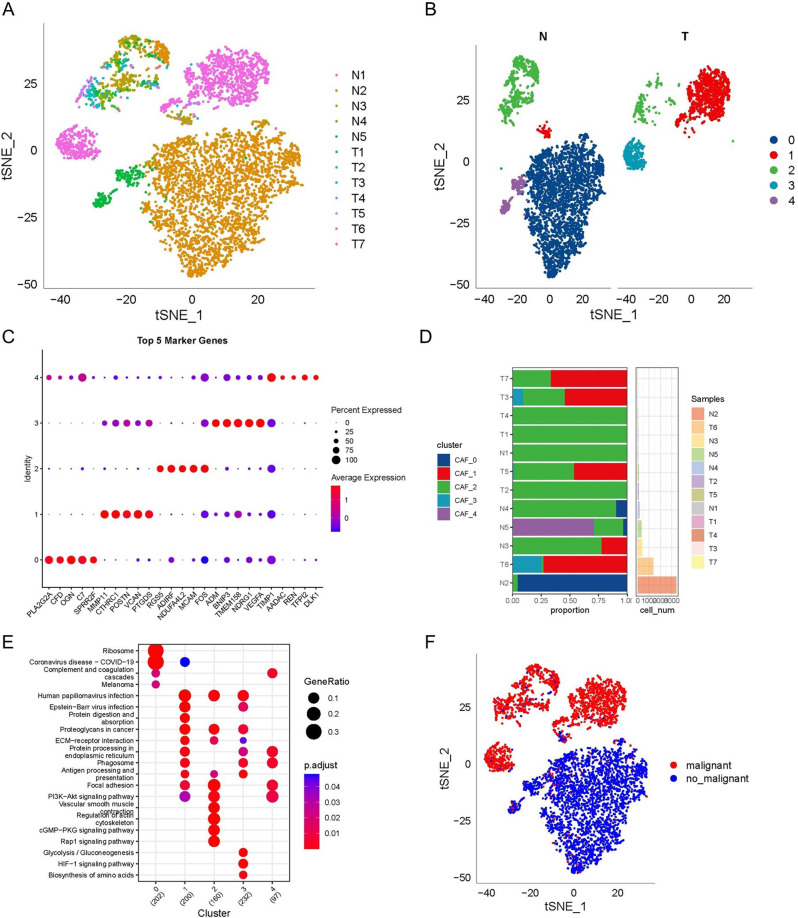
The identification of CAF clusters based on scRNA seq data of OC patients. (**A**) TSNE plot of the distribution of 12 samples; (**B**) TSNE plot of the distribution of five fibroblasts after clustering; (**C**) dot plot of the top 5 marker gene expression of subgroups; (**D**) subgroups in cancer tissue and Proportion and cell number of adjacent tissue; (**E**) kegg enrichment analysis of 5 fibroblast subsets; (**F**) TSNE distribution map of malignant and non-malignant cells predicted by copykat package

After preliminary screening, the scRNA-seq data yielded 40,810 cells. Figure S1 displays the detailed results of data preprocessing. Additionally, dimensionality reduction and log-normalization yielded 16 subpopulations, and using four marker genes-NOTCH3, PDGFRB, FAP, and ACTA2-five CAFs groups were found (Figures S2A, B). The cells of 5 CAFs clusters were extracted for further clustering and dimensionality reduction. The same clustering algorithm was applied to the CAFs clusters, identifying of five CAFs clusters (Figures S2C, D). None of the five CAFs subpopulations showed expression of the epithelial cell-specific gene, thereby validating the reliability of CAFs detection (Figure S3). The TSNE plot for the whole set of 12 distributions is presented in Fig. 1A. Consequently, five CAFs clusters were established and then analyzed (Fig. 1B). Overall, 1476 DEGs were found across the five CAFs clusters, and Fig. 1C displays the distribution of the five leading DEGs (detected as the gene markers of CAF clusters) throughout the five clusters. Moreover, Fig. 1D depicts the distribution of the five clusters per cohort. The KEGG analysis findings in Fig. 1E illustrated the enrichment of these DEGs in several different pathways, including the cGMP-PKG signaling pathway, PI3K-Akt signaling pathway, focal adhesion, etc. Furthermore, 1533 cancer and normal cells are distributed throughout the five CAFs clusters based on their CNV features (Fig. 1F).

### Expression profiles of cancer-associated pathways in CAFs

**Fig. 2:**
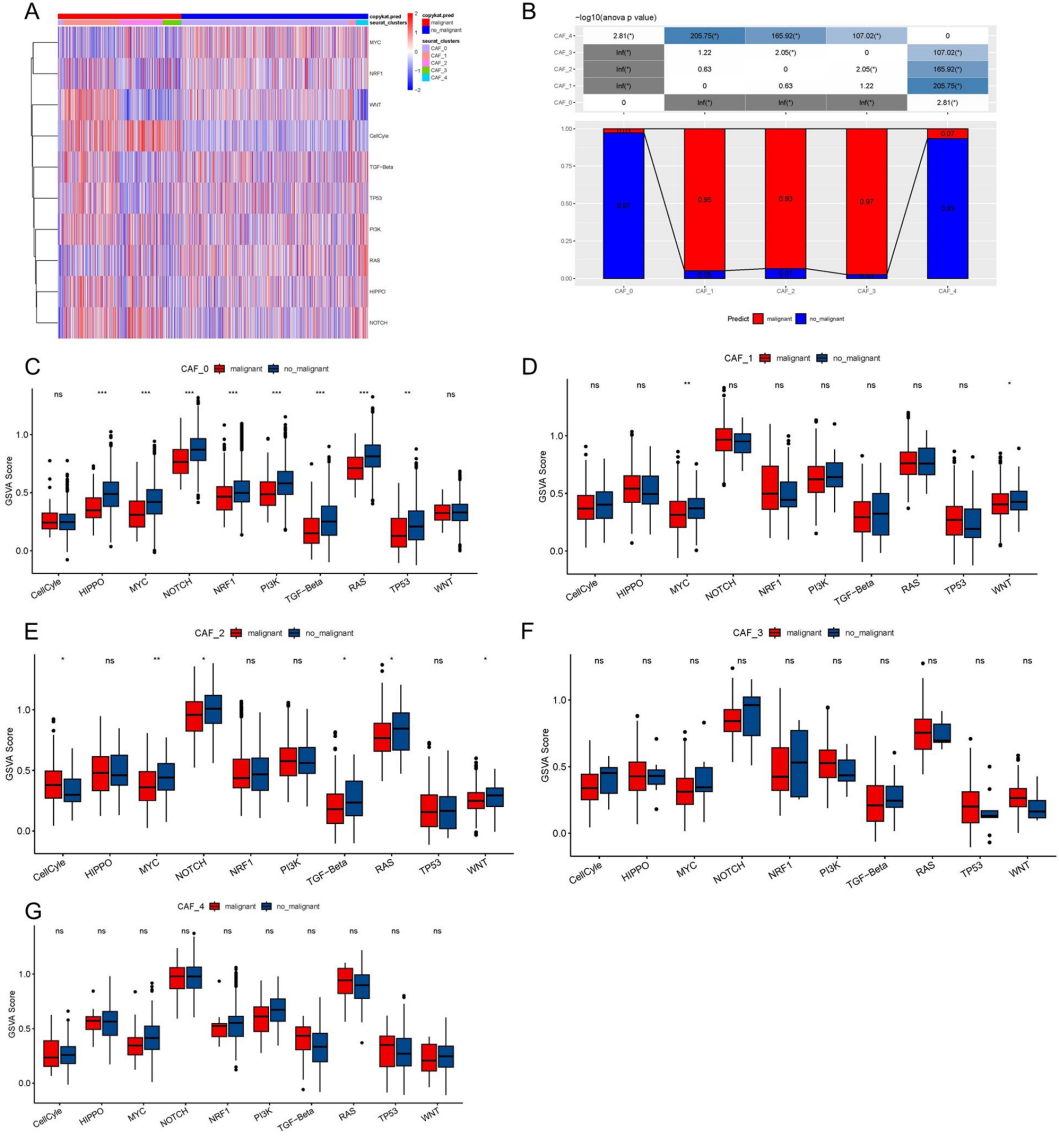
The characteristics of tumor-related pathways in CAF clusters. (**A**) Heatmap of 10 tumor-related pathway scores enriched in CAF cells; (**B**) Comparison of CAF clusters in malignant and non-malignant cells; Comparison of GSVA score of each pathway between malignant and non-malignant cells in CAF_0 (C), CAF_1 (D), CAF_2 (E), CAF_3 cluster (F), and CAF_4. (*P < 0.05; **P < 0.01; ***P < 0.001; and ****P < 0.0001). ns, not significant

**Fig. 3:**
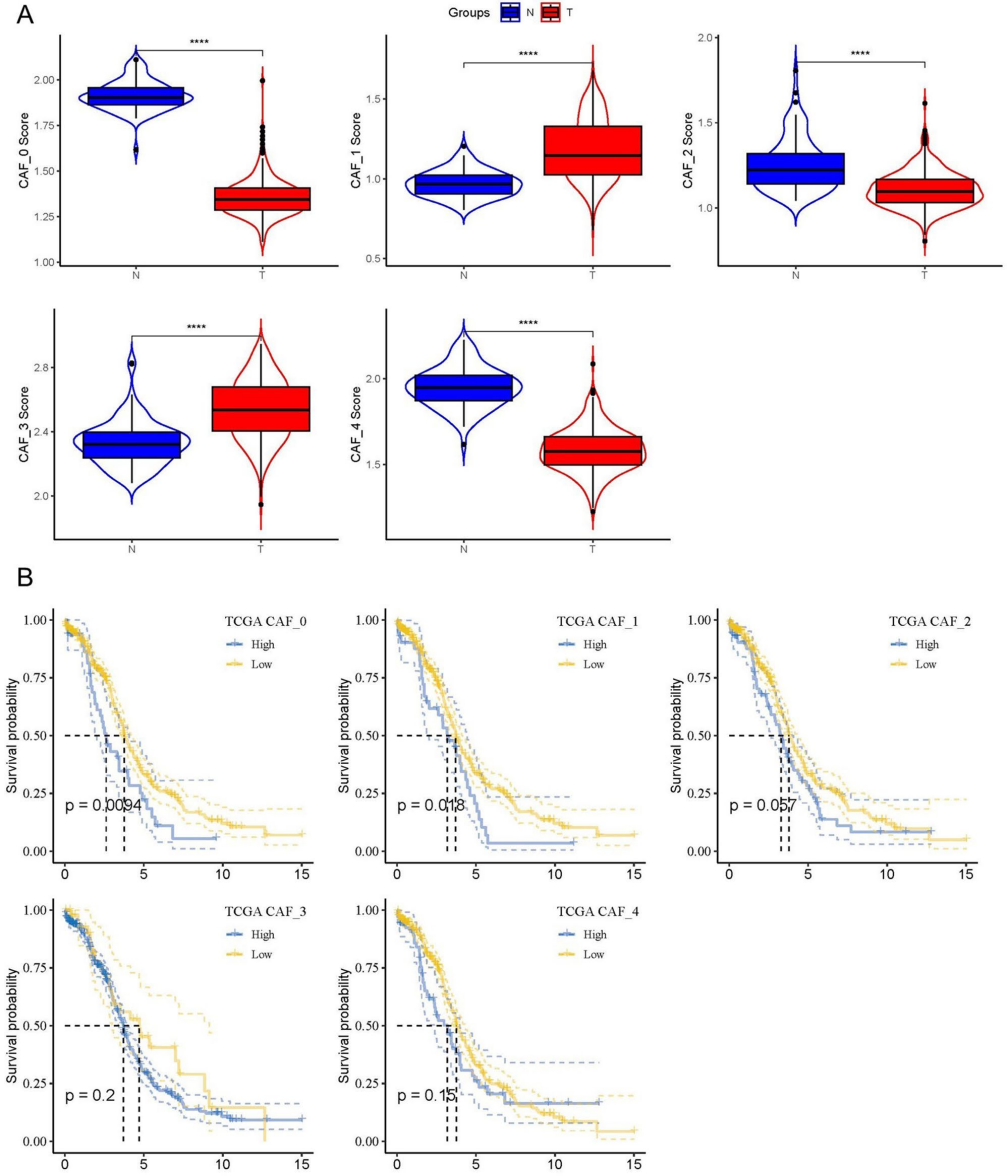
The associations between the five CAF cluster and prognosis of OC patients. (**A**) Comparison of five CAF scores in cancer and normal tissues; K-M curves of the high and low CAF score groups in the five CAF cluster (**B**). **P < 0.01, ****P < 0.0001

We analyzed the features of 10 tumor-related pathways in the five CAFs clusters to clarify the links between the clusters and tumor development. Figure 2A depicts the GSVA scores of the 10 tumor-related pathways across the various CAFs clusters. Notably, CAF_1, CAF_2, and CAF_3 had a significantly greater proportion of cancer cells than the other two clusters (Fig. 2B). Additionally, we compared cancerous and non-cancer cells within each CAF cluster using GSVA scores for the 10 tumor-related pathways and found some modest variations (Fig. 2C–G). We initially computed the ssGSEA score of the marker genes of each CAF cluster using the TCGA dataset (Fig. 1C displays the five most significant DEGs from CAF clusters) to identify any associations of the CAFs clusters with prognosis. As shown by the data, tumor samples scored considerably higher on the CAF_1 and CAF_3 clusters in contrast with normal samples, whilst other CAFs clusters showed the reverse pattern, with greater scores in normal tissues compared to tumors (Fig. 3A). The OC samples from the TCGA dataset were classified into high- and low-CAF score groups as per the optimal cut-off value analyzed by the survminer R package. Samples with higher CAFs scores fared poorly in both the CAF_0 and CAF_1 clusters in contrast with those in the low-CAF score group, whereas there was no correlation between the CAF_2, CAF_3, and CAF_4 clusters and a poor outcome in OC individuals (Fig. 3B–F).

### Discovery of CAFs-associated hub genes

**Fig. 4:**
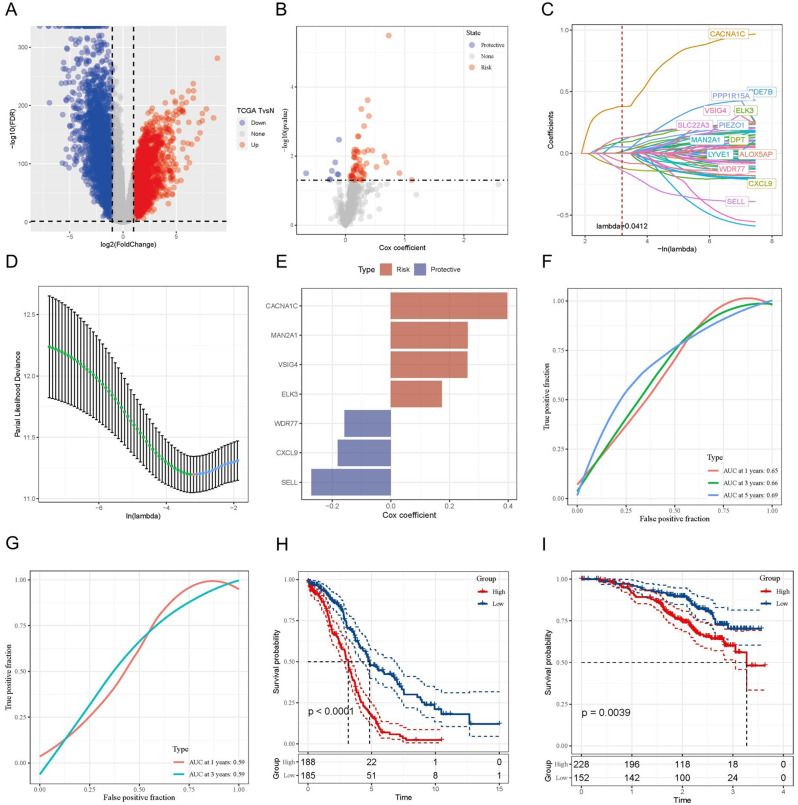
Identification of the hub predictive genes to construct a risk signature. (**A**) Volcano plot of differentially expressed genes of cancer and normal tissues in TCGA cohort; (**B**) Volcano plot of prognosis-related genes identified from univariate Cox regression analysis; (**C**) The trajectory of each independent variable with lambda; (**D**) Plots of the produced coefficient distributions for the logarithmic (lambda) series for parameter selection (lambda); (**E**) The multivariate Cox coefficients for each gene in the risk signature. (**F**) and (**G**) ROC curves of risk model constructed by 6 genes in TCGA cohort and GEO cohort; (**H**) and (**I**) K-M curves of risk model constructed by 6 genes in TCGA cohort and GEO cohort

**Fig. 5:**
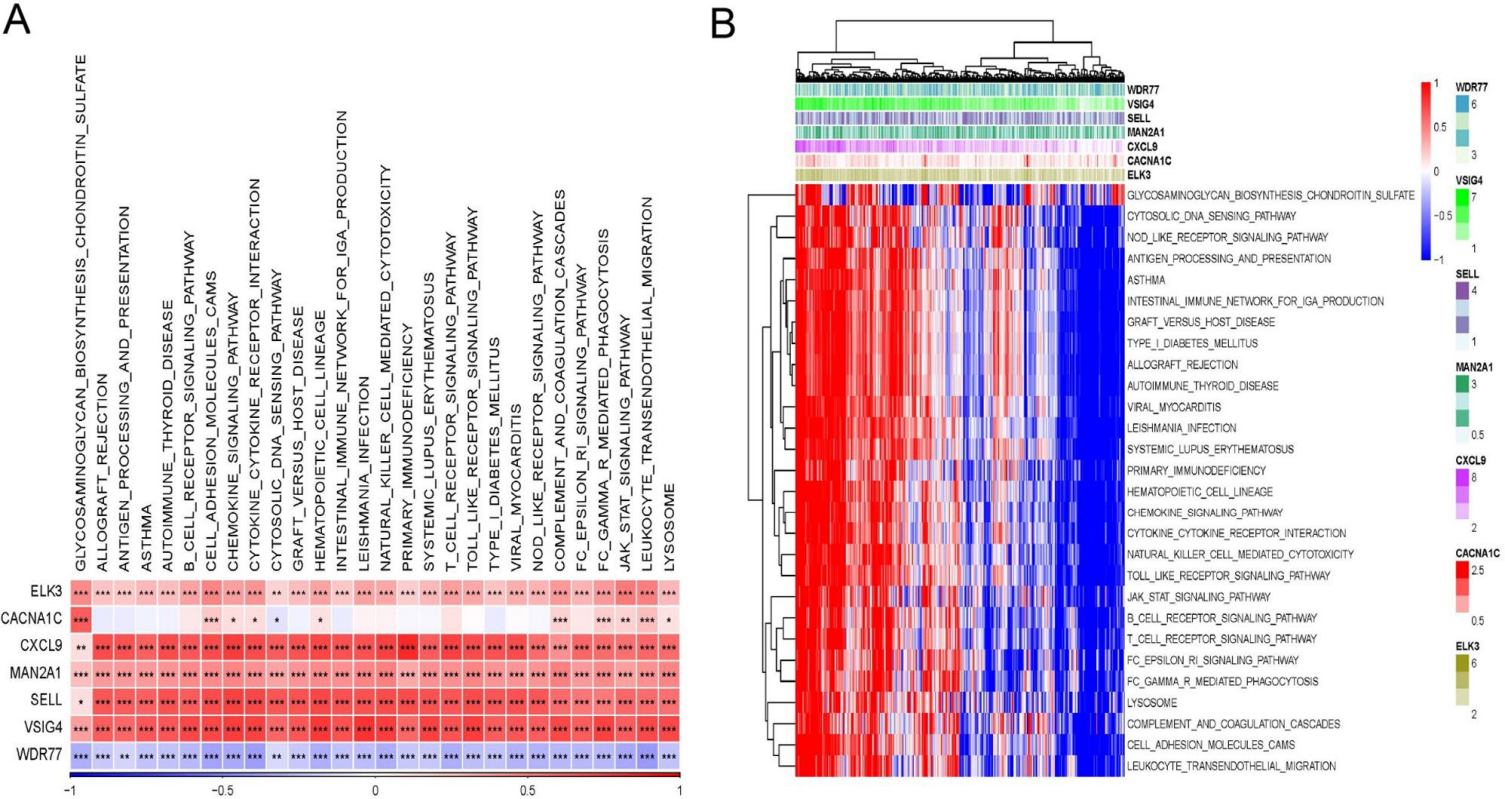
Identification of pathways that the risk genes involved in. (**A**) Gene-pathway correlation heatmap; (**B**) Enrichment score heatmap for key pathways. *P < 0.05, **P < 0.01, ***P < 0.001

We began by screening DEGs between tumor and normal tissues to develop a risk signature. Figure 5A demonstrates the identified 5808 DEGs, with 2769 DEGs showing an upregulation and 3039 DEGs showing a downregulation. In addition, 530 genes, in particular, were strongly correlated with the prognosis-associated CAFs clusters. Moreover, univariate analysis was undertaken to determine the prognostic significance of each gene and 66 genes was shown to be prognostic genes (Fig. 4A, B). After conducting Lasso Cox regression analysis to minimize the number of genes, only 14 remained with a lambda value of 0.0412 (Fig. 4C, D). Following the execution of multivariate analysis using the stepwise regression approach, we ultimately selected seven genes to construct the risk signature, including WD repeat domain 77 (WDR77), V-set, and immunoglobulin domain containing 4 (VSIG4), selectin L (SELL), mannosidase alpha class 2 A member 1 (MAN2A1), C-X-C motif chemokine ligand 9 (CXCL9), calcium voltage-gated channel subunit alpha1 C (CACNA1C), and ETS transcription factor ELK3 (ELK3) (Fig. 4E). The ultimate equation for the 7-gene signature is as follows: risk score = (0.174*ELK3) + (0.397*CACNA1C) +(-0.181*CXCL9) + (0.262*MAN2A1) + (-0.271*SELL) + (0.262*VSIG4) + (-0.158*WDR77). After applying the z-mean normalization, we determined each sample’s risk score and categorized them as either high or low risk predicated on that score. In the TCGA dataset, the AUC values for the model for 1- to 5-year survival varied from 0.65 to 0.69. However, in the GEO dataset, they varied from 0.65 (Fig. 4F, G). The Kaplan-Meier (KM) survival analyses illustrated that high-risk patients exhibited considerably worse survival status than those at low-risk in both the TCGA and GEO datasets (Fig. 4H, I).

### Analysis of mutations and pathways in the hub genes

The next step involved analyzing the SNV mutations in each of the seven genes used to develop the risk signature. A larger number of samples were found to contain SNV mutations in CACNA1C, VSIG4, CXCL9, ELK3, and MAN2A1, but no SNV variants were found in SELL or WDR77 (Figure S4A). We examined the probability that these important genes would co-occurrence along with the top 10 highly mutated genes. Figure S4B shows that whereas mutations in the aforementioned 5 genes did not show a statistically significant likelihood of co-occurrence, mutations in CSMD3 and CACNA1C did. We discovered that relatively few samples showed gain/loss of CNV in any of the 7 genes (Figure S4C). We investigated the links between the risk genes and various molecular signatures of OC to comprehend the nature of these links. The findings indicated that Homologous Recombination Defects, Fraction Altered, and the Number of Segments were strongly negatively correlated with ELK3, while CXCL9 and SELL were considerably positively associated with these same measures (Figure S4D). We also examined the possible pathways linked to each risk gene. Finally, as illustrated in Fig. 5A and B, the JAK-STAT, the Toll-like receptors receptor, the chemokine signaling, etc., were among the 28 pathways substantially linked to these seven genes.

### Association of hub genes with immunity

Based on our findings, we found that ELK3, CXCL9, MAN2A1, SELL, and VSIG4 all had positive associations with the stromal, immune, and estimate scores, whereas WDR77 had negative associations with all three (Figure S5A). We then evaluated the three scores among groups that were differentiated by their median expression levels. Results demonstrated a considerable variation between the high-expression and low-expression groups regarding the immune score for the ELK3, CACNA1C, CXCL9, MAN2A1, SELL, and VSIG4 genes, with the former exhibiting significant upregulation (Figure S5B). Strong inverse correlations were observed between M0 macrophages and CXCL9, MAN2A1, SELL, and VSIG4 (Figure S5C). Furthermore, analysis of correlations indicated that CXCL9, SELL, and VSIG4 exhibited a substantial positive correlation with most T cells (Figure S5D).

### Risk signature sensitivity to PD-L1 blockade immunotherapy

**Fig. 6:**
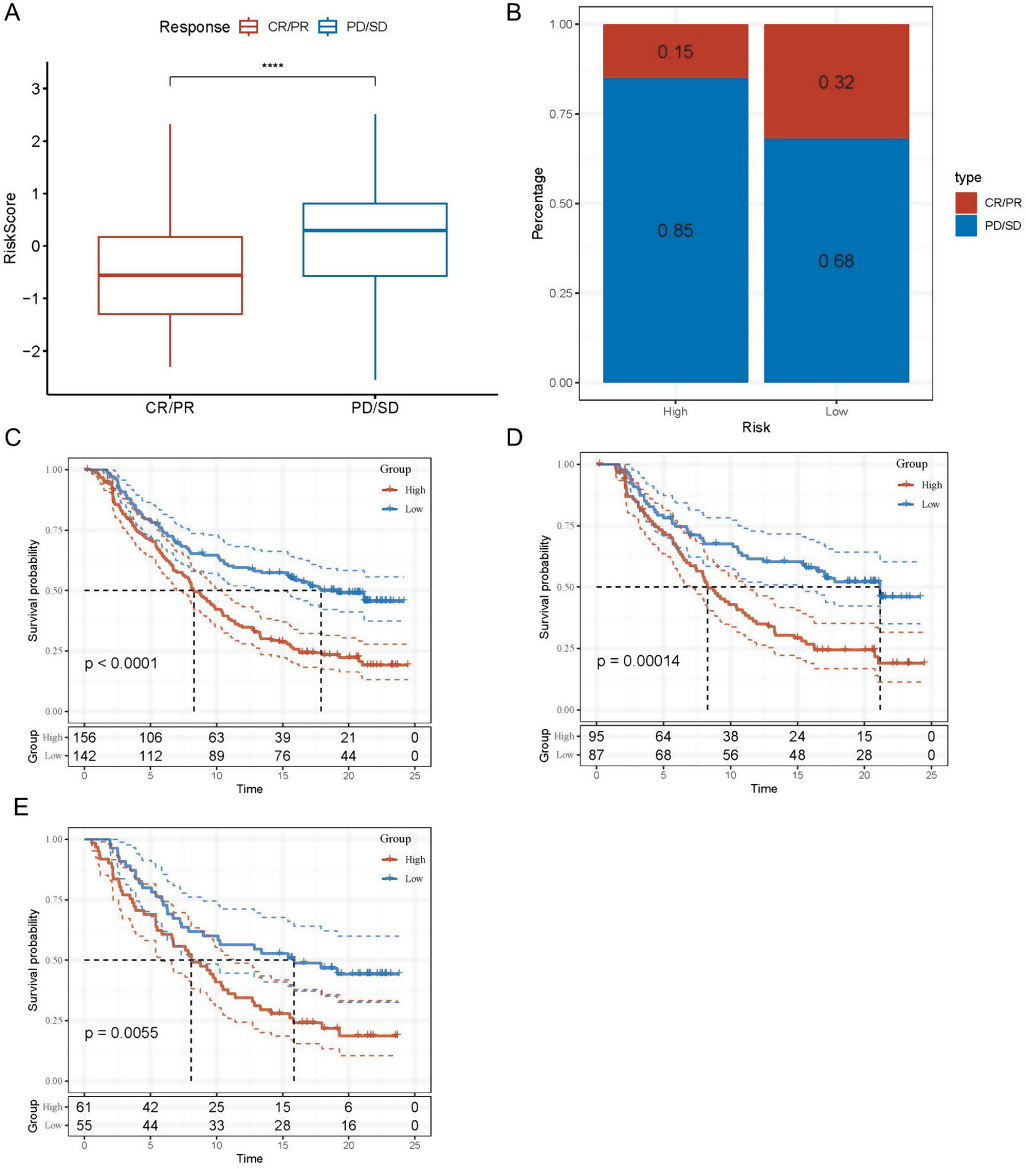
The responsiveness of risk score to PD-L1 blockade immunotherapy in IMvigor210 cohort. (**A**) Differences in risk scores among immunotherapy responses in the IMvigor210 cohort; (**B**) Distribution of immunotherapy responses among risk score groups in the IMvigor210 cohort; (**C**) Prognostic differences among risk score groups in the IMvigor210 cohort; (**D**) Prognostic differences between risk score groups in early stage patients in the IMvigor210 cohort; (**E**) prognostic differences between risk score groups in advanced patients in the IMvigor210 cohort. ****P < 0.0001

T-cell immunotherapy has made remarkable progress and is promising in treating cancer [25]. As a consequence, we evaluated the IMvigor210 risk signature’s predictive usefulness for immune-checkpoint therapy. Among the 348 patients in the IMvigor210 cohort, response to anti-PD-L1 receptor blockers varied, with outcomes ranging from complete response (CR) and partial response (PR) to stable disease (SD) and progressive disease (PD). As depicted in Fig. 6A, risk scores were higher in SD/PD patients as compared to CR/PR patients. A more significant proportion of people with SD/PD were found in the high-risk group compared to those in the low-risk category (Fig. 6B). Compared to the high-risk group, the low-risk group in the IMvigor210 cohort saw more favorable clinical outcomes and had substantially improved overall survival (OS) (Fig. 6C). Figure 6D shows a substantial difference in survival duration across various risk groups for patients in Stages I and II but not for those in Stages III and IV (Fig. 6E). Evidence from this study revealed that the risk score was more accurate for individuals at an earlier stage.

### Detection of independent risk variables and formulation of a risk model

**Fig. 7:**
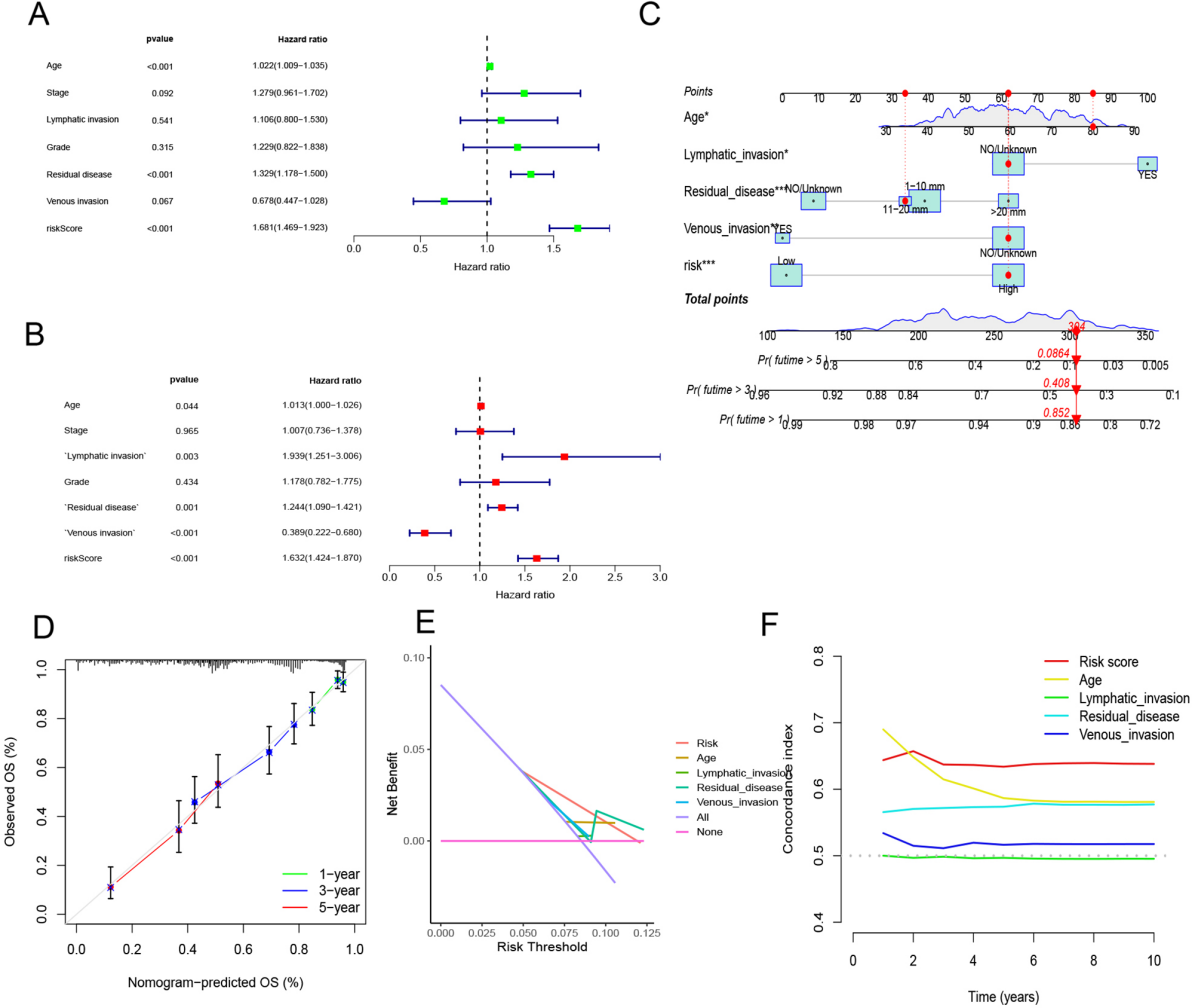
The development of a nomogram for predicting the prognosis of OC. (**A**, **B**) Univariate and multivariate Cox analysis of risk score and clinicopathological characteristics; (**C**) Nomogram model integrating the risk score and stage was constructed; (**D**) Calibration curves for 1, 3, and 5 years of nomogram; (**E**) Decision curve for nomogram; (**F**) Comparison of predictive capacity of clinicopathological features and the nomogram using time-ROC analysis. ***P < 0.001

Through univariate and multivariate analyses, we incorporated the clinicopathological features and risk score to enhance the risk signature’s predictive performance power. The risk signature emerged as the most influential prognostic predictor of OC in a multivariate model [hazard ratio (HR) = 1.632, 95% confidence interval (CI): 1.424–1.870, *P* < 0.001] (Fig. 7A, B). Consequently, the variables displayed in Fig. 7C (age, lymphatic invasion, residual disease, venous invasion, and risk score) were used to develop a nomogram. The nomogram’s ability to accurately predict real-world survival rates was proved via a calibration plot (Fig. 7D). Figure 7E further demonstrates that DCA found the nomogram to be more discriminative than the risk score and stage in identifying high-risk patients. Among the TCGA dataset, the AUC values of the risk score and nomogram were shown to be elevated in contrast with those of any other indicator, as evidenced by timeROC analysis (Fig. 7F).

### Key gene validation in CCLE databases

**Fig. 8:**
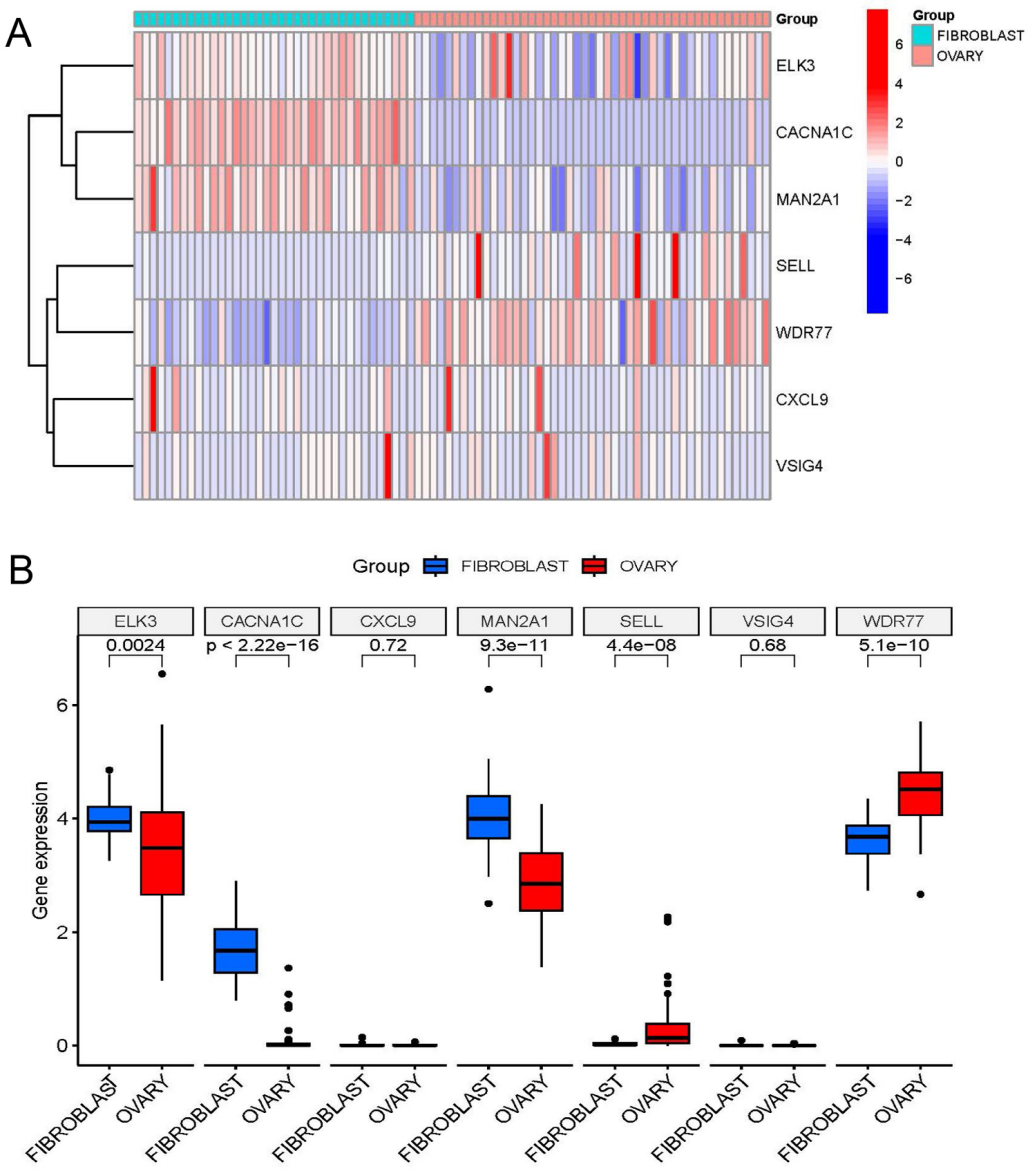
Differential expression of the 7 genes in OC in CCLE database. (**A**, **B**) The level of ELK3, CACNA1C, CXCL9, MAN2A1, SELL, VSIG4, and WDR77 in most ovarian cancer cell lines based on the CCLE database

We used the CCLE repository to confirm that fibroblast cell lines exhibited elevated mRNA expression levels of numerous genes (MAN2A1, CACNA1C, and ELK3) in contrast with OC cell lines (Wilcoxon test, all *p* < 0.001; Fig. 8A and B).

## Discussion

CAFs play a critical role in promoting the growth of OC cells by inducing tumor cell proliferation, angiogenesis, and immune suppression [26]. Studies have shown that CAF-secreted IL-8 can enhance OC stemness and malignancy, while exosomes from omental CAFs can increase peritoneal metastasis [27]. The gene GLIS1, which is upregulated in metastatic CAFs, can also promote OC cell migration and invasion [28]. However, much is still to be learned about the role of CAF-related genes in OC, and many researchers have focused on the impact of single genes. By studying gene signatures associated with CAFs, it may be possible to better understand the mechanisms behind OC progression and develop more targeted treatment strategies.

In this work, we analyzed the diversity of CAFs and conducted a comprehensive characterization and categorization of CAFs of OC using scRNA-seq data. The TME was divided into five CAFs clusters, each of which had unique characteristics and may have helped regulate some aspect of TME biology. A growing body of research has established the predictive significance of a CAF-associated gene signature in OC. Our findings showed that a score calculated from DEGs for the five clusters consistently illustrated that two clusters strongly correlate with the prognosis of OC individuals. Furthermore, CAF’s predictive performance could be attributed to the variations in WNT and NOTCH pathways we observed across CAFs groups. OC onset and progression may be enhanced by inhibiting apoptosis and promoting cell proliferation and differentiation via the stimulation of the WNT signaling pathway [29]. Additionally, The Notch signaling pathway is proven as a prominent component of OC implicated in the proliferation, migration, invasion, and treatment resistance [30].

Numerous studies have demonstrated that increased CAFs can act as an unfavorable prognostic factor in OC patients. Within the context of fibroblast biology and the tumor microenvironment in OC, CAFs, a group of non-immune-related tumor cells, may actively contribute to the proliferative, migratory, and metastatic capacities of tumor cells. Based on the high predictive value of two CAFs clusters, we have developed a CAF-based risk signature comprising seven genes. Of note, one of these genes, CXCL9, which acts as a ligand of CXCR3, has been reported to have a controversial role in tumor initiation and progression, exhibiting both positive and negative prognostic values depending on the type of tumor [31]. Interestingly, patients with OC who display elevated levels of CXCL9 have shown significantly higher relapse-free survival rates than those with low levels [32]. In response to Ras signaling, the transcriptional inhibitor ELK3 is transformed into a transcriptional activator by the phosphorylation of extracellular signal-regulated kinase 1/2 (ERK1/2) [33]. ELK3 overexpression has been observed in both OC cell lines and human malignancies [34]. CACNA1C, which encodes the alpha-1 subunit of a voltage-dependent calcium channel, has been linked to the modulation of cell adhesion, collagen fibril organization, cell-matrix adhesion, cell response to amino acid stimulation, and negative control of cell proliferation [35]. Previous research has shown a significant decrease in CACNA1C expression levels in OC tissues compared to healthy tissues [36]. The Golgi enzyme MAN2A1 is essential for transforming high mannose into a complex N-glycan structure to complete the glycosylation of protein membranes [37]. When fused with FER, MAN2A1 transforms into an oncogene; around 80% of prostate cancer patients with MAN2A1-FER have exhibited a dismal clinical prognosis according to earlier studies [38]. VSIG4, a novel macrophage protein linked to the B7 family, can inhibit T cell activation and may be involved in the onset and progression of cancer [39]. By suppressing the activity of complement pathways or T cells and promoting the development of regulatory T cells, VSIG4 can maintain immune system homeostasis, thereby inhibiting the progression of immune-induced inflammatory diseases but promoting cancer advancement [40]. However, there is a scarcity of functional validation for the seven genes implicated in the CAFs of OC, necessitating further investigations of the 7 CAFs markers.

New research indicates that CAFs may enhance tumor development through their interactions with the TME [41]. Our analysis revealed that six the prediction genes positively correlated with the immune score. In contrast, while one risk gene had a negative correlation, suggesting possible interactions between these genes and the TME in OC. This highlights the potential of these genes as treatment targets for OC. The TME comprises various immune cells that work together to create an anti-tumor immune response. CAFs can create an immunosuppressive TME that helps cancer cells evade immune surveillance by interacting with immune cells. Our research showed that the prognostic genes of the risk signature were positively correlated with many types of T cells, which play a critical role in tumor growth and are promising targets for immunotherapies such as ICI and CAR-T cell therapy [42]. The risk signature may also identify patients most likely to respond to immunotherapies.

Furthermore, the results demonstrated that a CAFs-based signature might predict a patient’s response to anti-PD-L1 immunotherapy. Our findings provide valuable insights into CAFs’ role in reshaping the cancer niche and immune state in TME. Nevertheless, further studies are warranted to clarify the significance of CAFs-TME crosstalk in OC and its potential for use in OC immunotherapy.

However, it is important to note that our study has several limitations. First, we utilized retrospective data from public repositories to establish the CAFs clusters and risk signature. Therefore, it will be imperative to validate its effectiveness in additional prospective and multicenter studies involving OC patients in the future. Second, the CAF-based risk signature was only assessed for its potential prognostic value; further investigation is needed to elucidate the underlying mechanisms by which this signature contributes to the initiation and progression of OC.

## Conclusion

Overall, the findings of our study suggest that CAFs play a critical role in the onset and progression of OC, and the CAF-based risk signature could function as a useful prognostic tool for predicting the survival outcomes of OC patients. Moreover, the signature can also potentially aid in identifying patients who are most likely to benefit from immunotherapies. However, further studies are needed to validate the effectiveness of the signature in larger, multi-center OC cohorts and to elucidate the underlying mechanisms and biological processes involved in the CAFs-TME crosstalk in OC.

### Electronic supplementary material

Below is the link to the electronic supplementary material.

**Supplementary Material 1. Figure S1.** The results of re-process of scRNA-seq data of OC. (A) The relationship between mitochondrial genes and the amount of UMI/mRNA, the relationship between UMI and the amount of mRNA; (B) The relationship among mRNA, UMI, mitochondrial content, and rRNA content of each sample before filtering; (C) The relationship among mRNA, UMI, mitochondrial content, and rRNA content of each sample after filtering; (D) The sample distribution map of PCA dimensionality reduction and the anchor point map of PCA.

**Supplementary Material 2. Figure S2.** The clustering of CAF populations and dimensionality reduction. (A) Distribution of subpopulations after clustering of all cells; (B) TSNE map of fibroblast marker gene expression; (C) Distribution of subpopulations after re-clustering of fibroblasts; (D) TSNE diagram of marker expression in five CAF clusters.

**Supplementary Material 3. Figure S3.** The expression of EPCAM in four CAF clusters.

**Supplementary Material 4. Figure S4.** The characteristics of mutations of the genes included in the risk signature. (A) Waterfall diagram of SNV mutations of 7 key genes; (B) Colinearity and mutual exclusion analysis of key genes and the 10 most mutated genes in tumors; (C) CNV mutations (gain, loss, none) of 6 key genes; (D) Correlation heatmap of 6 key genes with Aneuploidy Score, Homologous Recombination Defects, Fraction Altered, Number of Segments, and Nonsilent Mutation Rate.

**Supplementary Material 5. Figure S5.** The relationship between the risk genes and immune landscape. (A) The correlation matric of the risk genes and stromal score, immune score, and estimate score. (B) Comparison of high and low expression of key genes and immune score; (C) Correlation between key genes and immune cell score predicted by CIBERSORT analysis; (D) Comparison of high and low expression of key genes with 22 immune cell scores (*P < 0.05; **P < 0.01; ***P < 0.001; and ****P < 0.0001.)

## Data Availability

The datasets used and/or analyzed in this study are available from the corresponding author on reasonable request.

## Abbreviations

CAFs: Cancer-associated fibroblasts
CAR: chimeric antigen receptor
CACNA1C: calcium voltage-gated channel subunit alpha1 C
CCLE: Cancer Cell Line Encyclopedia
CNV: copy number variations
CXCL9: C-X-C motif chemokine ligand 9
DCA: Decision curve analysis
DEGs: differentially expressed genes
ECM: extracellular matrix
FDR: false discovery rate
ELK3: ETS transcription factor ELK3
GEO: Gene Expression Omnibus
HPA: Human Protein Atlas
HR: hazard ratio
ICIs: immune-checkpoint inhibitors
KM: Kaplan-Meier
Lasso: least absolute shrinkage and selection operator
MAN2A1: mannosidase alpha class 2 A member 1
OC: ovarian cancer
OS: overall survival
ROC: receiver operating characteristic
scRNA-seq: single-cell RNA sequencing
SNV: single-nucleotide variant
SELL: selectin L
TME: tumor microenvironment
TCGA: The Cancer Genome Atlas
TSNE: t-distributed stochastic neighbor embedding
VSIG4: V-set, and immunoglobulin domain containing 4
WDR77: WD repeat domain 77
