# Supplementary figures and images for: Integration of single-cell RNA-seq and bulk RNA-seq data to construct and validate a cancer-associated fibroblast-related prognostic signature for patients with ovarian cancer
Source: J Ovarian Res. 2024 Apr 16;17:82. doi: 10.1186/s13048-024-01399-z (PMC11020192; doi:10.1186/s13048-024-01399-z)

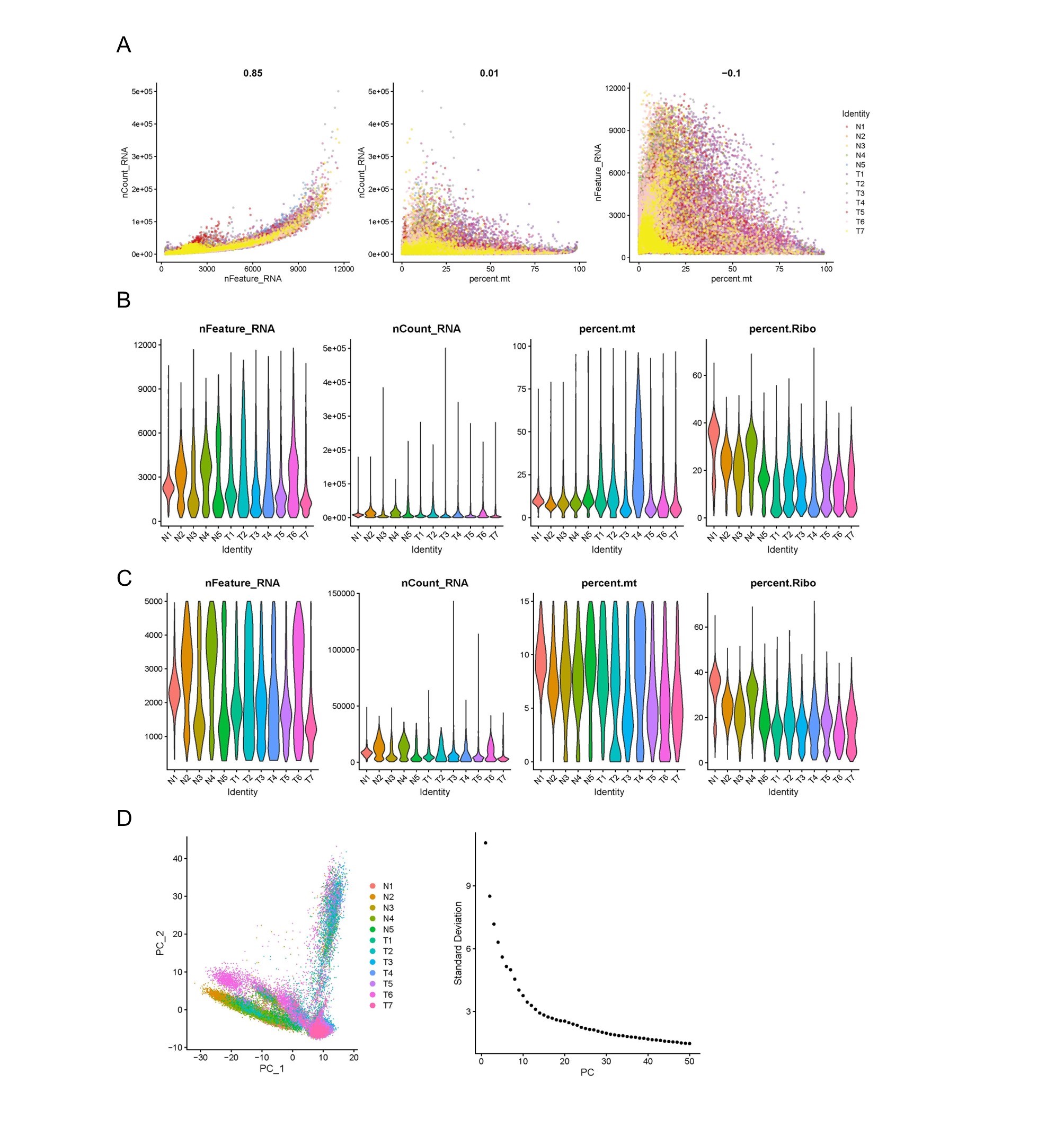

Supplement: Supplementary file 1 — Supplementary Material 1. Figure S1. The results of re-process of scRNA-seq data of OC. (A) The relationship between mitochondrial genes and the amount of UMI/mRNA, the relationship between UMI and the amount of mRNA; (B) The relationship among mRNA, UMI, mitochondrial content, and rRNA content of each sample before filtering; (C) The relationship among mRNA, UMI, mitochondrial content, and rRNA content of each sample after filtering; (D) The sample distribution map of PCA dimensionality reduction and the anchor point map of PCA. [file 13048_2024_1399_MOESM1_ESM.jpeg]

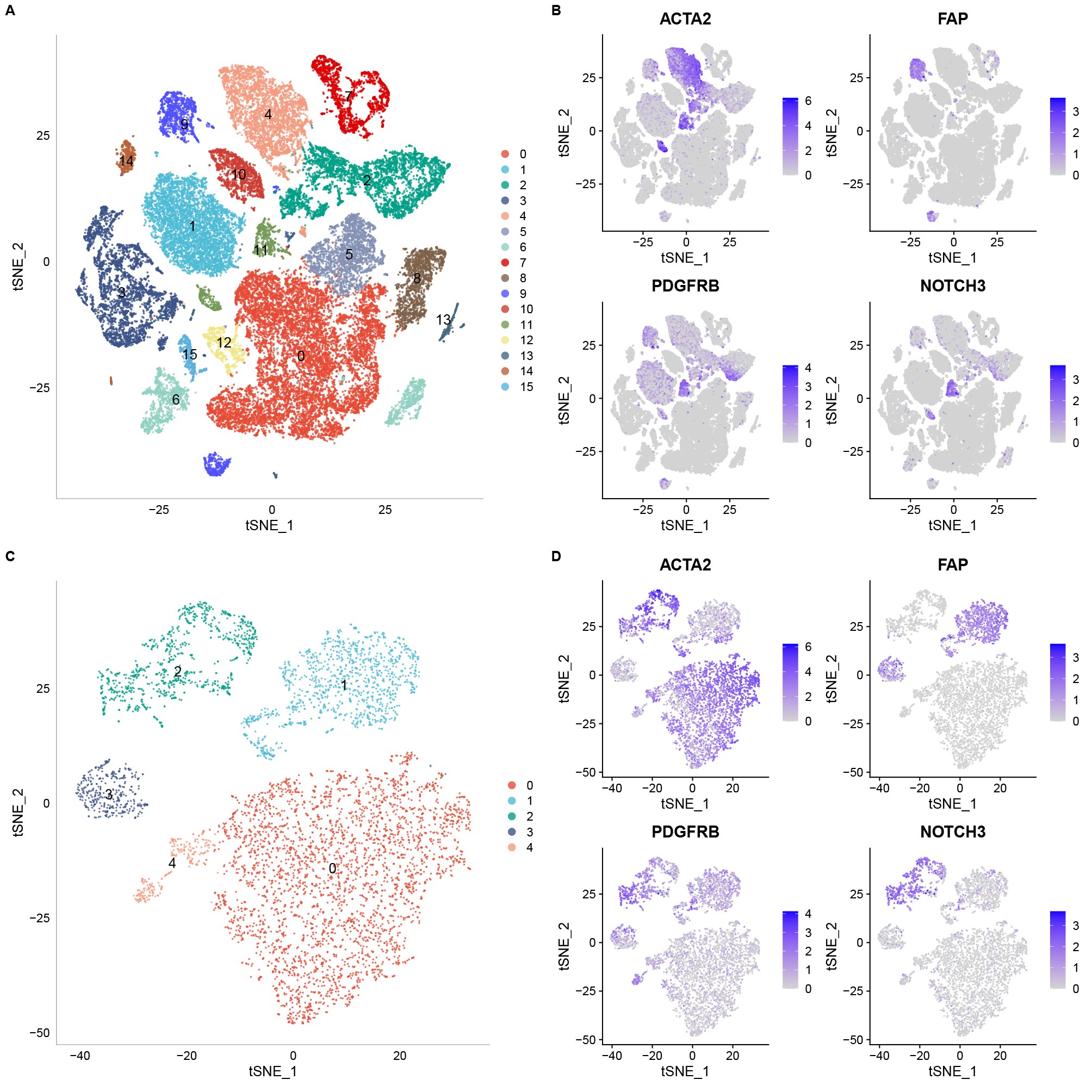

Supplement: Supplementary file 2 — Supplementary Material 2. Figure S2. The clustering of CAF populations and dimensionality reduction. (A) Distribution of subpopulations after clustering of all cells; (B) TSNE map of fibroblast marker gene expression; (C) Distribution of subpopulations after re-clustering of fibroblasts; (D) TSNE diagram of marker expression in five CAF clusters. [file 13048_2024_1399_MOESM2_ESM.jpeg]

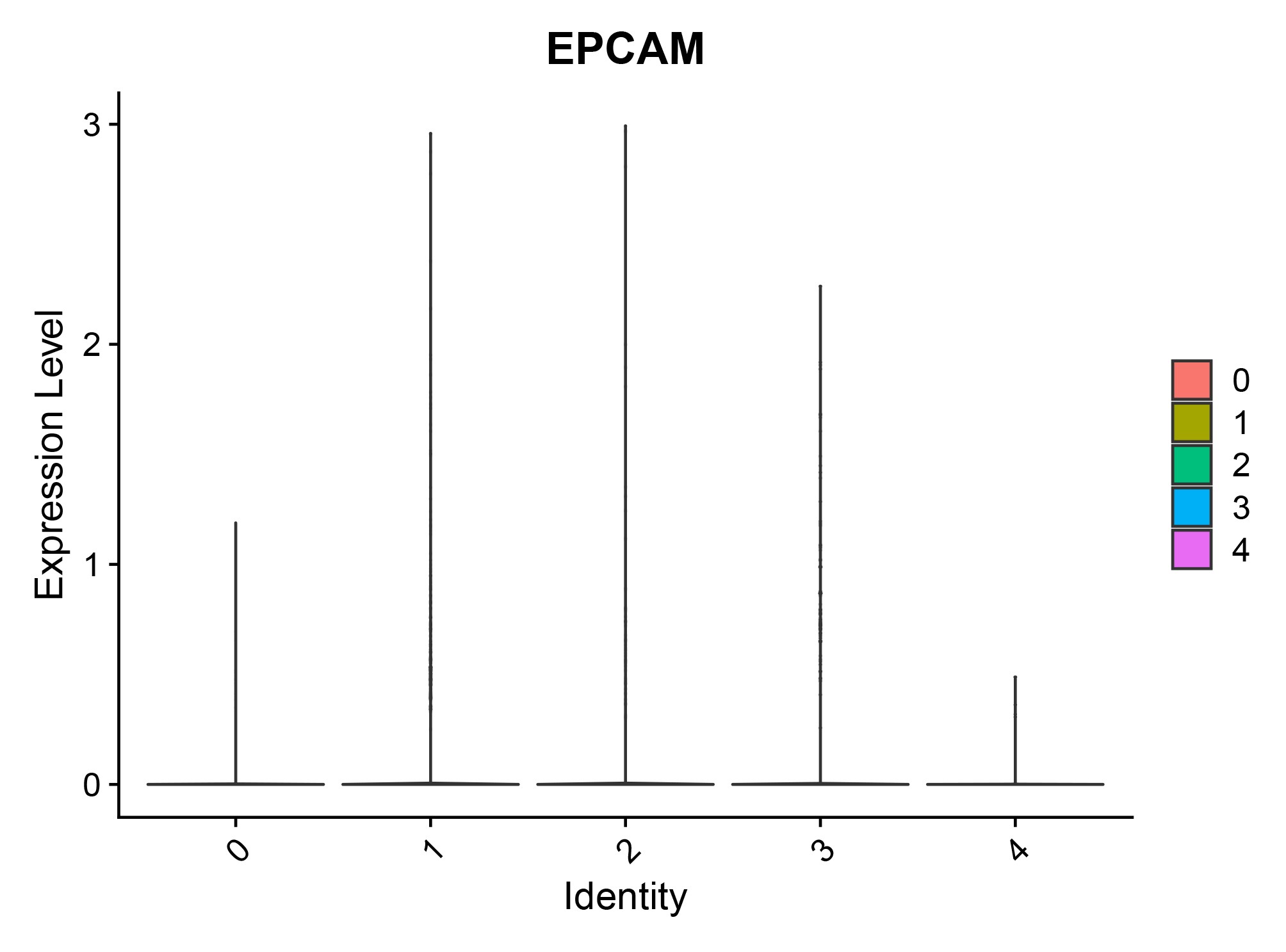

Supplement: Supplementary file 3 — Supplementary Material 3. Figure S3. The expression of EPCAM in four CAF clusters. [file 13048_2024_1399_MOESM3_ESM.jpeg]

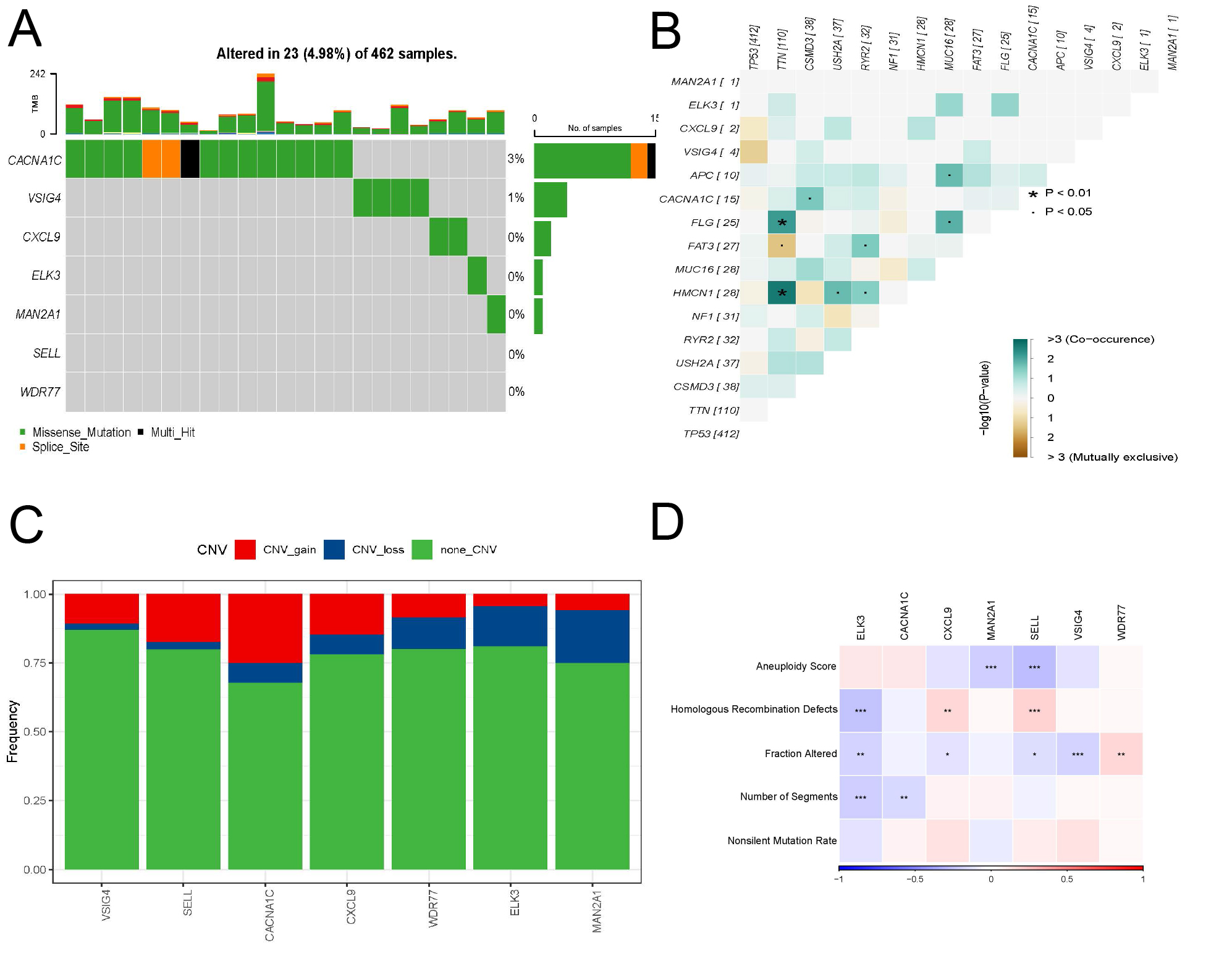

Supplement: Supplementary file 4 — Supplementary Material 4. Figure S4. The characteristics of mutations of the genes included in the risk signature. (A) Waterfall diagram of SNV mutations of 7 key genes; (B) Colinearity and mutual exclusion analysis of key genes and the 10 most mutated genes in tumors; (C) CNV mutations (gain, loss, none) of 6 key genes; (D) Correlation heatmap of 6 key genes with Aneuploidy Score, Homologous Recombination Defects, Fraction Altered, Number of Segments, and Nonsilent Mutation Rate. [file 13048_2024_1399_MOESM4_ESM.jpeg]

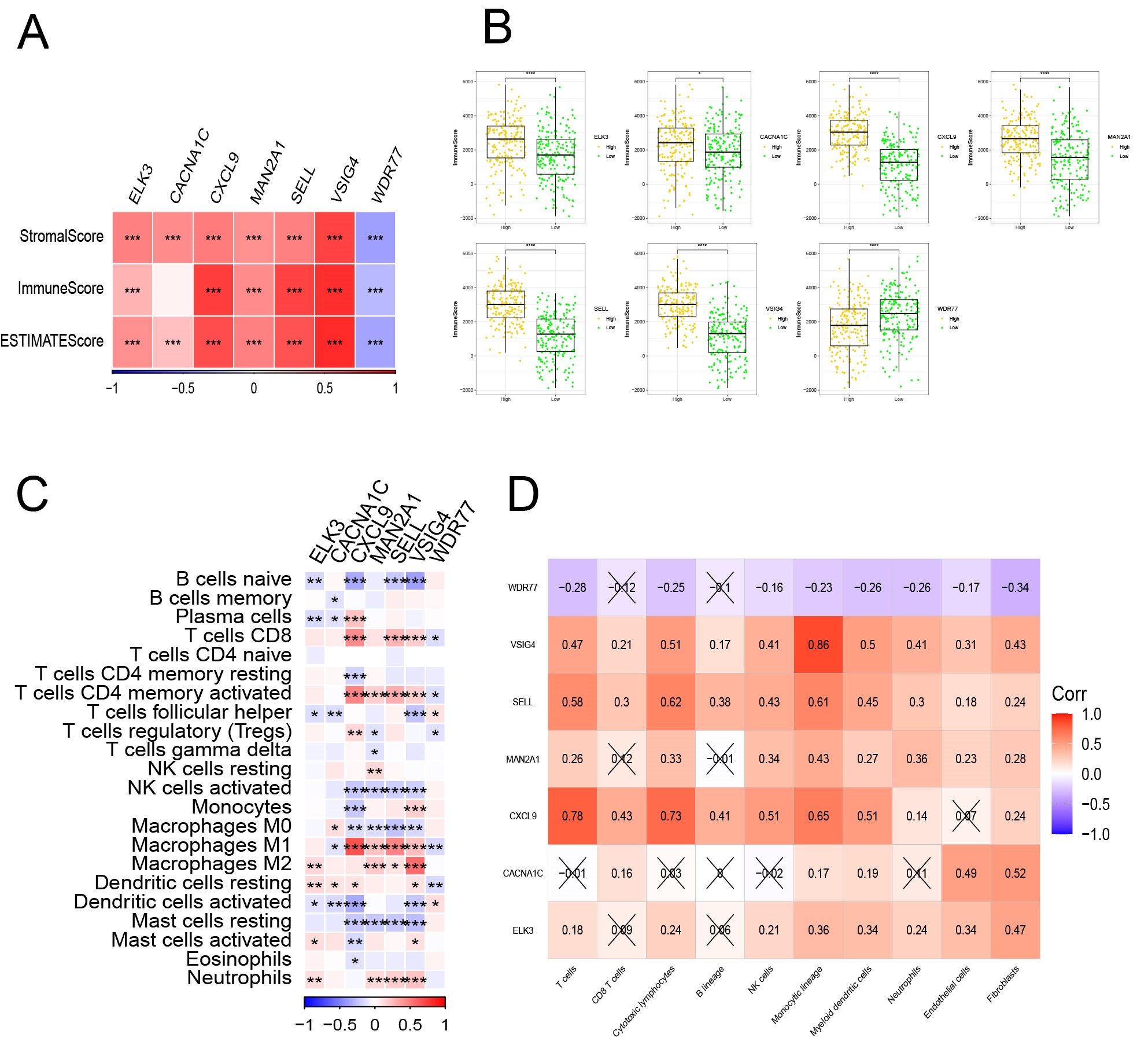

Supplement: Supplementary file 5 — Supplementary Material 5. Figure S5. The relationship between the risk genes and immune landscape. (A) The correlation matric of the risk genes and stromal score, immune score, and estimate score. (B) Comparison of high and low expression of key genes and immune score; (C) Correlation between key genes and immune cell score predicted by CIBERSORT analysis; (D) Comparison of high and low expression of key genes with 22 immune cell scores (*P < 0.05; **P < 0.01; ***P < 0.001; and ****P < 0.0001.) [file 13048_2024_1399_MOESM5_ESM.jpeg]
